# Recent Developments in the Applications of Fingerprinting Technology in the Food Field

**DOI:** 10.3390/foods11142006

**Published:** 2022-07-07

**Authors:** José S. Câmara, Sonia Medina, Rosa Perestrelo

**Affiliations:** 1 CQM—Centro de Química da Madeira, NPRG, Campus Universitário da Penteada, Universidade da Madeira, 9020-105 Funchal, Portugal; rmp@staff.uma.pt; 2 Departamento de Química, Faculdade de Ciências Exatas e da Engenharia, Campus Universitário da Penteada, Universidade da Madeira, 9020-105 Funchal, Portugal; 3 Department of Food Science and Technology, Research Group on Quality, Safety and Bioactivity of Plant Foods, CEBAS (CSIC), Campus Espinardo, 30100 Murcia, Spain; soniamedes@gamil.com

In recent years, the concerns and demands by consumers for the high quality and safety for natural and processed plant-based and animal foods has increased significantly. The main demands are directed towards shelf life, adulterations, food origin, and composition related with potential health benefits.

Food contamination and food adulteration requires continued efficient vigilance, since it might occur with some regularity. In this context, the development of high resolution and sensitive analytical and detection techniques for identification and/or quantification of typical components, adulterants, and/or contaminants of foods is required. Currently, a combination of well-established and recognized high-resolution analytical methods, including mass spectrometry techniques (GC-MS, LC-MS/MS, MALDI-TOFMS), in addition to modern spectroscopic approaches based on NMR (1H; 13C), IR, and sensor technologies, amongst others, seems to be the most promising approach to food fingerprinting. This will provide a useful strategy to establish its composition and certify its integrity (food traceability, safety, quality, and authenticity), constituting a useful platform on fraud detection, adulterations, and contaminations.

The food’s fingerprint constitutes an important support for mapping food characteristics with potential uses to evaluate its integrity, expressed on traceability and authenticity, determine its geographical origin, and identify contaminants among other undesirable compounds; this will improve consumer’s protection while giving more confidence, transparency, and better knowledge of foods.

In this context, we proposed a relevant and emerging topic “*Recent Developments in the Applications of Fingerprinting Technology in the Food Field*” for a Special Issue (SI) in *Foods* journal, aiming to address relevant contributions on all aspects of food fingerprinting technologies, while constituting a useful platform to enable the food industry, governments, and regulatory entities to keep up-to-date with the most recent and innovative developments in production technologies and processes in addition to analytical fingerprinting techniques and recent advances in food science. Thus, this Special Issue includes eleven publications discussing different analytical approaches regarding with recent developments towards the applications of fingerprinting in the food field [1,2,3,4,5,6,7,8,9,10,11].

The paper by Câmara et al. [1] reviewed a deep insight into the health and applications potential of polyphenols. The chemical structure, classification, and biosynthesis are highlighted. The challenge of polyphenols bioavailability and bioaccessibility is explored as well as the useful environmental and industrial applications. Advanced and emerging extraction techniques are also discussed, and the high-resolution analytical techniques used for food bioactive compounds (FBCs) characterization, identification, and quantification are considered.

Since pork is the most consumed red meat in Cypriot population, Kyriakides et al. [2] reported a dietary exposure assessment of veterinary antibiotics found in raw pork meat among children and adolescents in Cyprus, in a context of food safety. The study was based on the results of the occurrence of 45 residual antibiotics in raw pork meat samples in Cyprus between 2012 and 2017 in combination with data on the consumption of pork meat on children and adolescents taken from the latest demographic. The results indicate that antibiotic residues in pork meat of inland production are below the acceptable daily intake and are of low risk to human health. Nevertheless, continuous exposure to low levels of antibiotic residues in respect to age vulnerability should be of a great concern. The fingerprint markers of PDO “Pera Rocha do Oeste”, a PDO Pear Native from Portugal, was established by Pedro et al. [3] based on classical physicochemical parameters (colour, total soluble solids (TSS), pH, acidity, ripening index, firmness, vitamin C, total phenols, protein, lipids, fibre, ash, other compounds including carbohydrates, and energy). Near infrared (NIR) spectroscopy was used as a complementary tool to assess the global variability of the samples [3].

In another manuscript, the feasibility of non-targeted UHPLC-HRMS fingerprints as chemical descriptors to address the classification and authentication of paprika samples was evaluated by Barbosa et al. [4]. Non-targeted UHPLC-HRMS fingerprints were obtained after a simple sample extraction method and C18 reversed-phase separation. Fingerprinting data based on signal intensities as a function of m/z values and retention times were registered in negative ion mode with a q-Orbitrap high-resolution mass analyzer, and the obtained non-targeted UHPLC-HRMS fingerprints subjected to unsupervised principal component analysis (PCA) and supervised partial least squares regression-discriminant analysis (PLS-DA) to study sample discrimination and classification. Zhu et al. [5] investigated the metabolome of raw meat from Jiulong yaks, focusing on specimens farmed and harvested locally using untargeted nuclear magnetic resonance spectroscopy (1H-NMR) as the analytical platform. Samples from longissimus thoracis, trapezius, triceps brachii, and biceps femoris muscles, with different prevalence’s of red and white fibers, were selected. A metabolic pathway analysis suggested that the main pathways differing among the muscles were connected to the turnover of amino acids [5].

To evaluate the changes on the aroma characteristics of shiitake mushrooms (Lentinus edodes) during hot-air drying, volatile compounds of *L. edodes* were analyzed by Qin et al., [6] using electronic nose, sensory evaluation, and purge and trap combined with gas chromatography-mass spectrometry (PT-GC-MS) at different timepoints of the drying process. The obtained results suggested that the unique flavor of dried mushrooms is mainly due to the activation of the enzymatic pool during the drying process, which act on lentinic acid to produce sulfur-containing heterocyclic compounds. We believe that this study enhances our understanding to the mushroom cultivation and processing industry to achieve an improvement in sensory quality and characterizations vs. possible fraudulent products.

Pleva et al. [7] studied the correlation between the heterocyclic amine (HCA) concentration and the color changes of the chicken breast with or without skin during grilling under open or closed conditions as a function of time and temperature. The concentration of the HCAs formed during grilling was measured by a validated LC–MS/MS method, whereas the color changes were determined by changes of CIELAB (International Commission on Illumination) values (L*, brightness; a*, redness; b*, yellowness), using a CHROMA METER CHR-400.

Non-targeted approaches relying on high-performance liquid chromatography with ultraviolet detection (HPLC-UV) fingerprints were evaluated by Nuñez et al. [8] to address coffee characterization, classification, and authentication by chemometrics. LC-UV data revealed good chemical descriptors for the classification of coffee samples by partial least squares regression-discriminant analysis (PLS-DA) according to geographic origin.

An interesting paper carried out by Fernandes et al. [9] reported the volatile signature of onions from different geographical regions of Madeira Island (Caniço, Santa Cruz, Ribeira Brava, and Porto Moniz) established by headspace solid-phase microextraction (HS-SPME/GC-qMS) and chemometric tools, showing that the volatile signature was affected by the geographical region of cultivation.

A comprehensive study performed by Park et al. [10] examines the interface between primary and secondary metabolites in oval- and rectangular-shaped Chinese cabbage (*Brassica rapa* ssp. *pekinensis*) using gas chromatography coupled with time-of-flight mass spectrometry (GC-TOFMS) and high-performance liquid chromatography (HPLC). This metabolomic study comprehensively describes the relationship between primary and secondary metabolites in the oval and rectangular cultivars of Chinese cabbage and provides useful information for developing strategies to enhance the biosynthesis of glucosinolates, phenolics, and carotenoids.

Finally, Katerinopoulou et al. [11] mapped the current state of research in authentication and certification of geographic origin of agricultural products as a useful tool towards the protection of high-quality foods, identifying emerging fields to the geographical origin of products.

As guest editors of this Special Issue, we would like to thank all the authors and co-authors for their excellent contribution, all reviewers for their effort in revising the manuscripts, as well as the editorial office of *Foods* journal for their generous help in organizing this Special issue.
